# Supplementary material for: Low-Energy Electron Induced Reactions in Metronidazole at Different Solvation Conditions
Source: Pharmaceuticals (Basel). 2022 Jun 2;15(6):701. doi: 10.3390/ph15060701 (PMC9227036; doi:10.3390/ph15060701)
Supplement: Supplementary file 1 [file pharmaceuticals-15-00701-s001.zip › pharmaceuticals-1728141-supplementary.pdf]

Additional comments to Table S1 and Figures S1-S4:

Since it is the mass of the anionic species identified experimentally, in most cases various products combinations (neutral species) are theoretically possible. The thresholds listed in Table S1 correspond to the combination of products (Fig. S1), where ΔG value was the lowest (for 0 eV experimental onset) or of the lowest deviation from experimental data.

Most of the fragment anions, except $NO_2^-$ (mass 46 u) and (Metro-$C_2H_4OH$)$^-$ (126 u), form after multiple bond breakage. One may also expect that the $OH^-$ anion arises from the single bond cleavage, but that would produce a threshold of 1.16 eV. Therefore, we suggest a bit more complicated neutral products (similar to these of ($NO_2$+$C_2H_3OH$)$^-$ (90 u)) that result in an exothermic threshold of –0.74 eV.

(Ring+H)$^-$ (66 u), (Ring+$CH_3$+H)$^-$ (81 u) and ($NO_2$+$C_2H_3OH$)$^-$ (90 u) anions require a bit more discussion as these are somehow connected. All three involve the product of 90 u mass, either as a radical or as an anion, arising from the recombination of $NO_2$ and $C_2H_4OH$ with one hydrogen abstracted. Interestingly, all three could involve different hydrogen abstractions (C6–H, C7–H and O8–H). For the (Ring+H)$^-$ (66 u), the abstraction of the O8–H yields the ΔG value of 2.76 eV (best corresponding to the experimental value of 2.69 eV), while C6–H or C7–H would give 2.35 and 2.18 eV respectively. For (Ring+$CH_3$+H)$^-$ (81 u) abstraction of C7–H produces the lowest ΔG value of –0.86 eV, two other positions being 0.17 eV (C6–H) and 0.58 eV (O8–H) higher. It is worth noting, that in this case all three values are negative, therefore equally corresponding to the experimentally observed 0 eV. Finally, for ($NO_2$+$C_2H_3OH$)$^-$ (90 u) the fragment is in the anionic state and C7–H abstraction makes the molecule unstable, while other two are connected with the calculated threshold of –1.09 eV (C6–H) or –0.35 eV (O8–H) – again both positions are possible, as the experiment gives us the 0 eV threshold.

For (Metro-$NO_2$-OH)$^-$ (108 u) and (Metro-$NO_2$-$CH_3$)$^-$ (110 u) two bonds break, initially producing two radicals ($NO_2$ and OH or $CH_3$). It is worth noting, that (probably due to the distance between these) for the first case $NO_2$ and OH combine to give $HONO_2$ molecule. If that is not the case we would not observe the threshold of 1.99 eV, but instead 2.90 eV. Similarly, from the fact that we observe the threshold of 2.77 eV and not 1.44 eV, we can conclude that $NO_2$ does not combine with $CH_3$ in case of (Metro-$NO_2$-$CH_3$)$^-$ (110 u).

While from the remaining, (Metro-CHOH-$NO_2$)$^-$ (95 u) and (Metro-$NO_2$-2H)$^-$ (123 u) are pretty straightforward, ($NO_2$+N+$C_2H_4OH$)$^-$ (105 u), $CN^-$ (26 u) and $CNO^-$ (42 u) formation involve more complicated mechanisms, including bond cleavage in the ring and further rearrangements, see Figure S2-S4. These are difficult to assess having only anion masses. For the first one, ($NO_2$+N+$C_2H_4OH$)$^-$ (105 u), depending on the rearrangements that could occur after the initial bond breaks we've found the thresholds energies ranging from –0.53 eV to even 3.85 eV. The most probable value of 1.20 eV (0.76 eV lower than the experimental value) also seems to be one of the least complicated. Similarly, for the $CN^-$ anion (26 u) we've found 0.74 eV (1.86 eV lower than the experimental value) that corresponds to pretty simple fragmentation scheme. The calculated $CNO^-$ (42 u) anion threshold is 0.78 eV lower than the experimental one and thus a possible scheme to explain the experimental result.

**Table S1.** The calculated thresholds and AEA of anions, as calculated at M06-2X/aug-cc-pvtz level of theory. All values shown in eV.

| Mass (u) | Reaction anionic product | M06-2X/aug-cc-pvtz | | | | | Experimental |
|---|---|---|---|---|---|---|---|
| | | | 1 atm | | *2.92x10⁻¹¹ atm* | | |
| | | AEA | 298.15 K | 360.15 K | 298.15 K | 360.15 K | |
| 17 | $OH^-$ | 1.66 | 2.03 | 1.52 | 0.16 | -0.74 | 0 |
| 26 | $CN^-$ | 4.07 | 2.50 | 2.25 | 1.25 | 0.74 | 2.60 |
| 42 | $CNO^-$ | 3.41 | 2.64 | 2.53 | 2.01 | 1.78 | 2.56 |
| 46 | $NO_2^-$ | 2.35 | 0.53 | 0.40 | -0.10 | -0.35 | 0 |
| 66 | $(Ring+H)^-$ | 3.02 | 4.52 | 4.27 | 3.27 | 2.76 | 2.69 |
| 81 | $(Ring+CH_3+H)^-$ | 2.40 | 0.03 | -0.11 | -0.60 | -0.86 | 0 |
| 90 | $(NO_2+C_2H_3OH)^-$ | 2.83 | -0.21 | -0.34 | -0.84 | -1.09 | 0 |
| 95 | $(Metro\text{-}CHOH\text{-}NO_2)^-$ | 1.83 | 1.37 | 1.13 | 0.12 | -0.38 | 0 |
| 105 | $(NO_2+N+C_2H_4OH)^-$ | 3.44 | 2.08 | 1.96 | 1.45 | 1.20 | 1.96 |
| 108 | $(Metro\text{-}NO_2\text{-}OH)^-$ | 1.96 | 2.88 | 2.74 | 2.25 | 1.99 | 2.15 |
| 110 | $(Metro\text{-}NO_2\text{-}CH_3)^-$ | 3.11 | 4.52 | 4.28 | 3.27 | 2.77 | 2.63 |
| 123 | $(Metro\text{-}NO_2\text{-}2H)^-$ | 2.26 | -0.39 | -0.59 | -1.64 | -2.09 | 0 |
| 126 | $(Metro\text{-}C_2H_4OH)^-$ | 3.64 | -0.58 | -0.72 | -1.21 | -1.47 | 0 |
| 171 | $Metro^-$ | 1.24 | | | | | 0 |

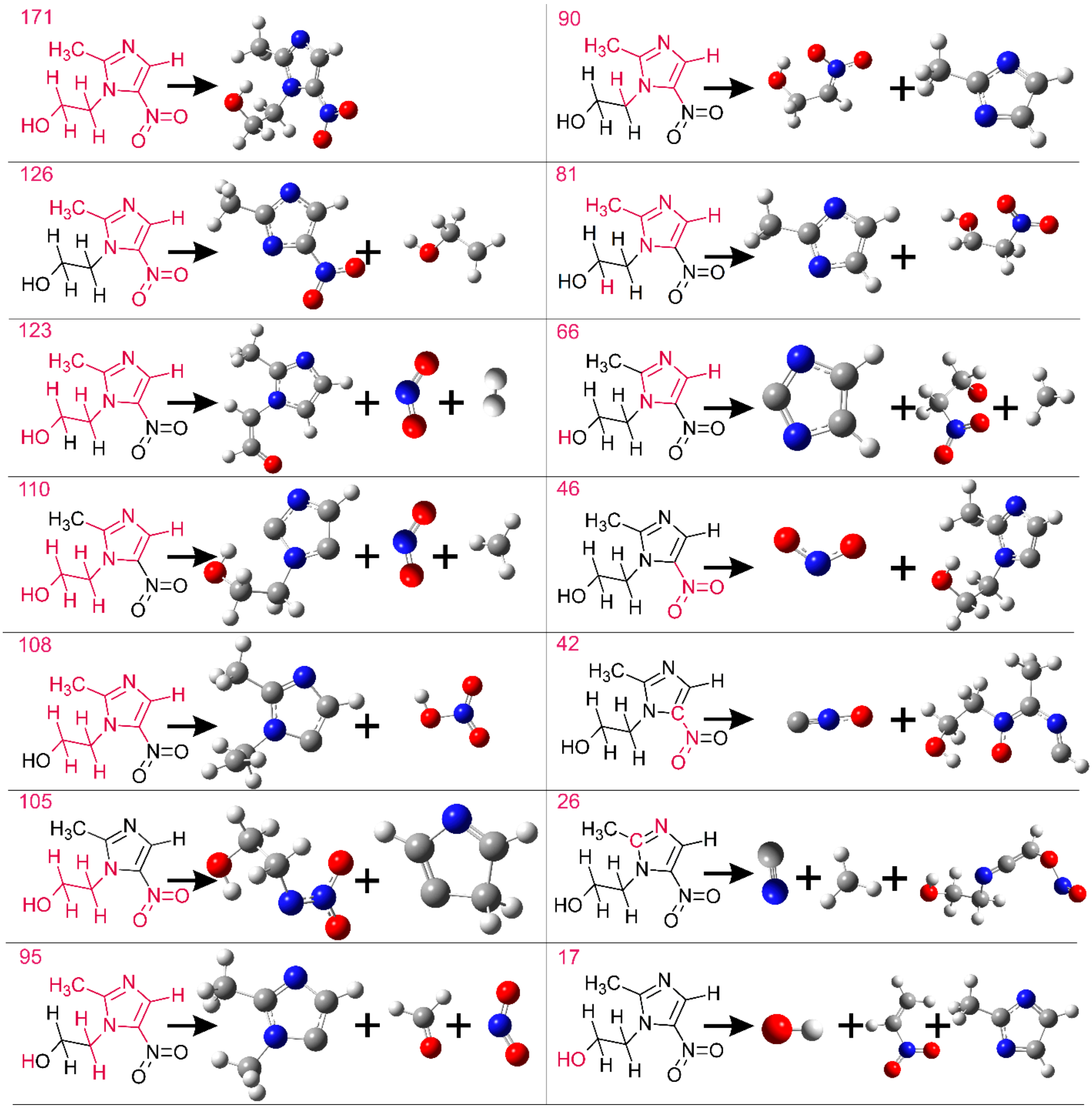


**Figure S1.** Possible dissociation pathways for the observed anions upon electron attachment to Metro.

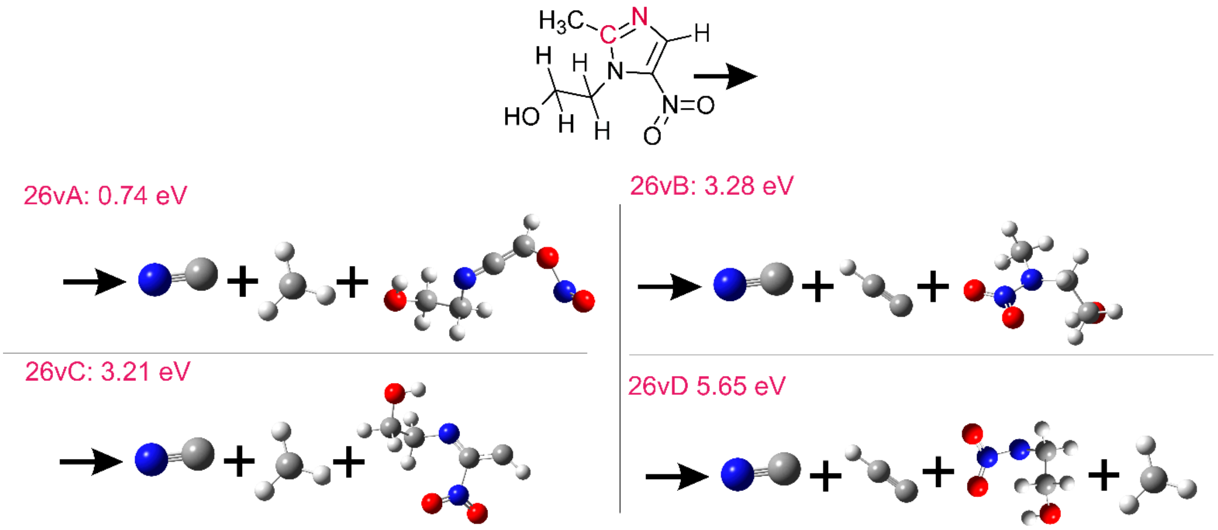


**Figure S2.** Possible dissociation pathways leading to $CN^-$ upon electron attachment to Metro.

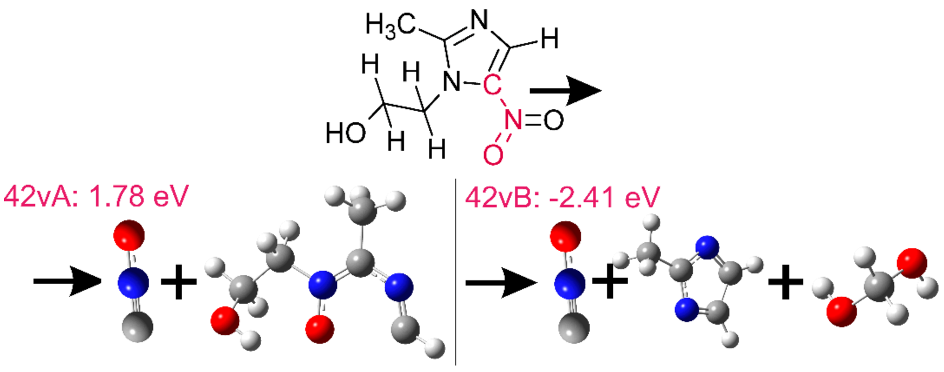


**Figure S3.** Possible dissociation pathways leading to $CNO^-$ upon electron attachment to Metro.

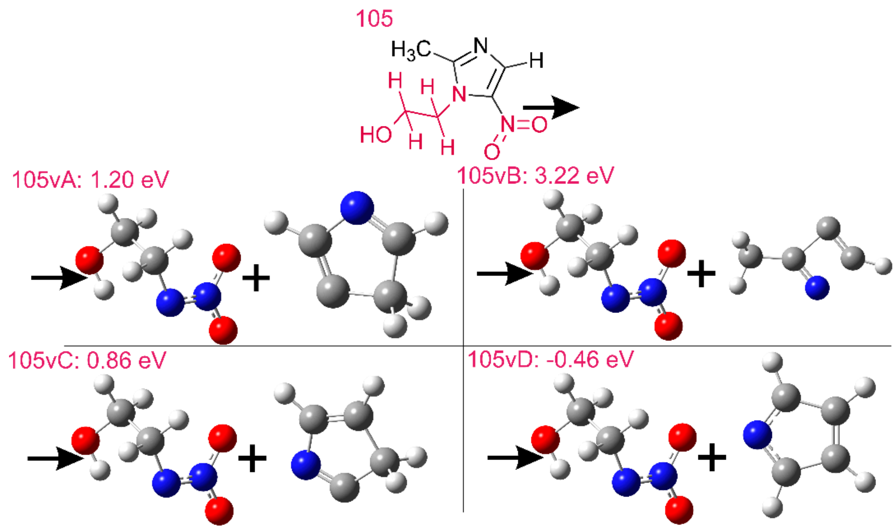


**Figure S4.** Possible dissociation pathways leading to $(NO_2{+}N{+}C_2H_4OH)^-$ upon electron attachment to Metro.

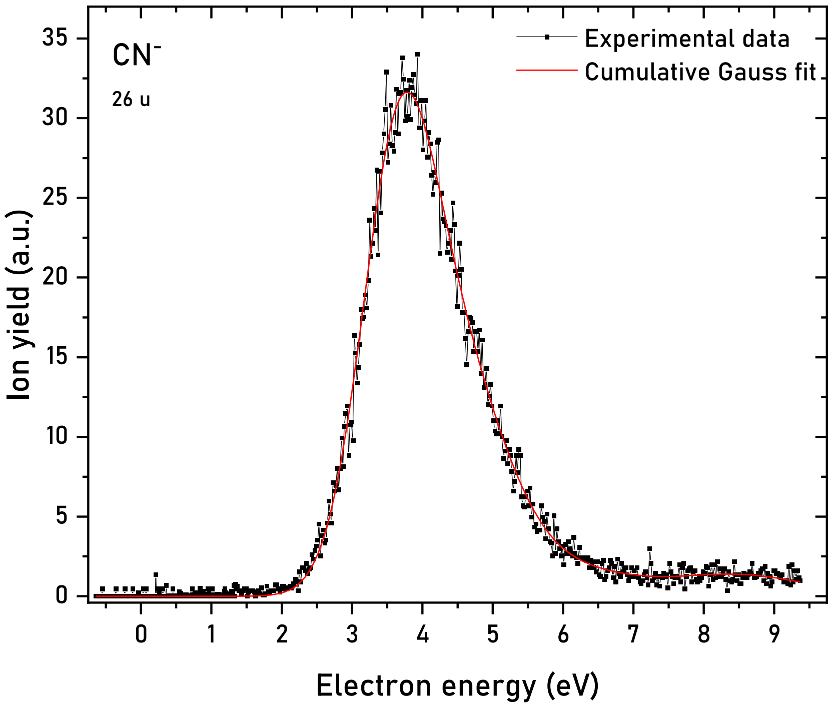


**Figure S5.** Anion efficiency curve for $CN^-$ formed upon electron attachment to isolated Metro.

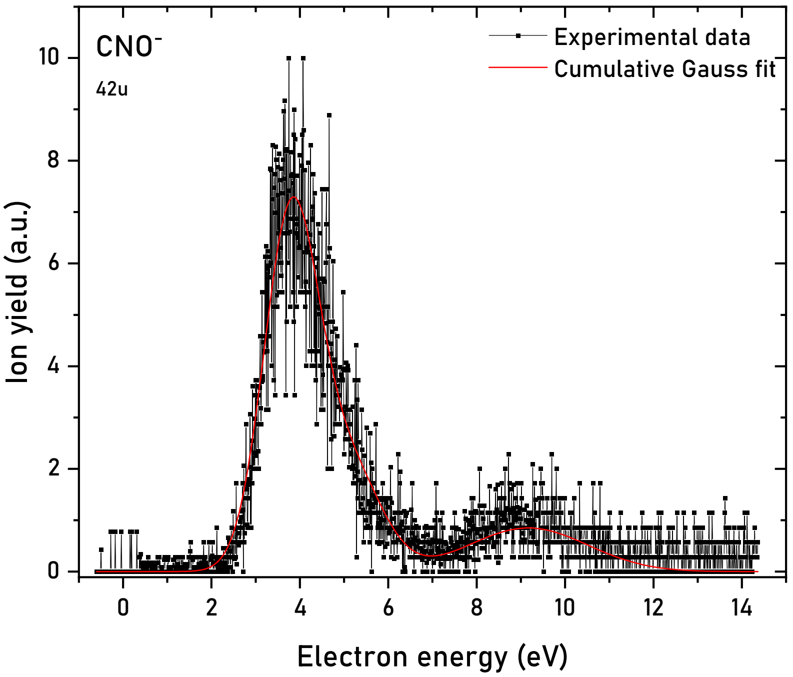


**Figure S6.** Anion efficiency curve for $CNO^-$ formed upon electron attachment to isolated Metro.

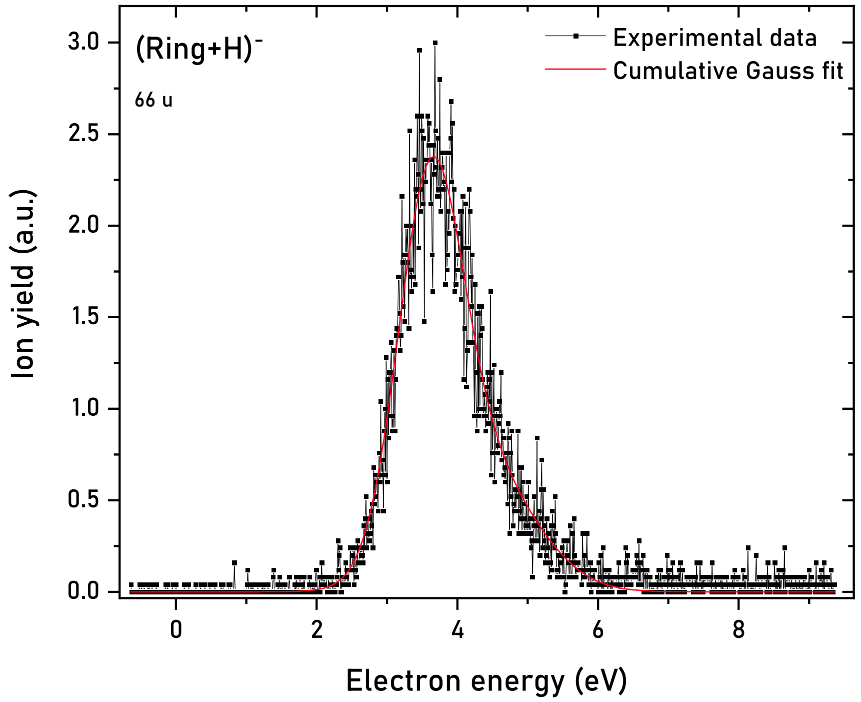


**Figure S7.** Anion efficiency curve for (Ring+H)$^{-}$ formed upon electron attachment to isolated Metro.

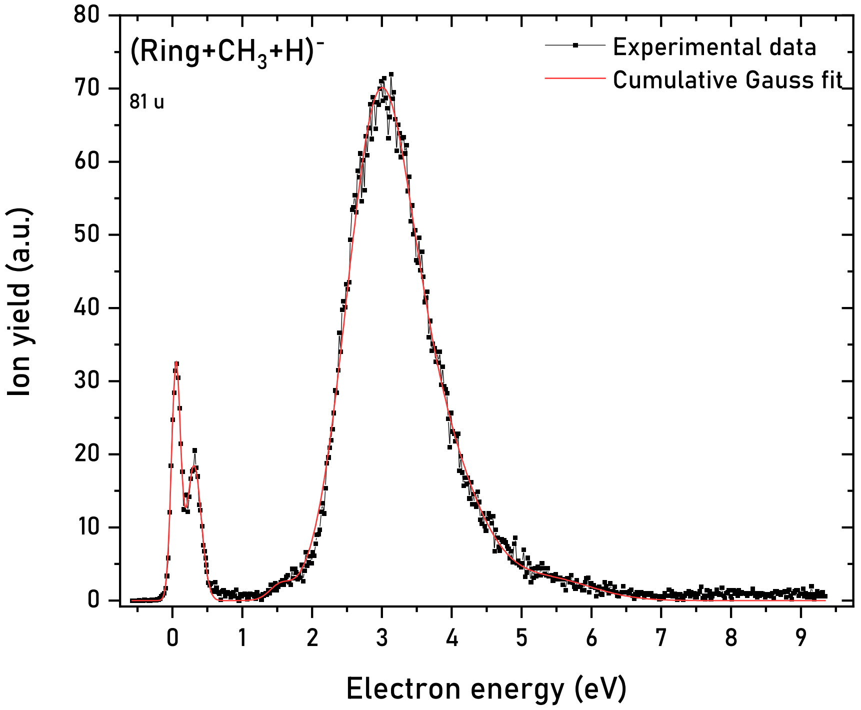


**Figure S8.** Anion efficiency curve for (Ring+$CH_3$+H)$^{-}$ upon electron attachment to isolated Metro.

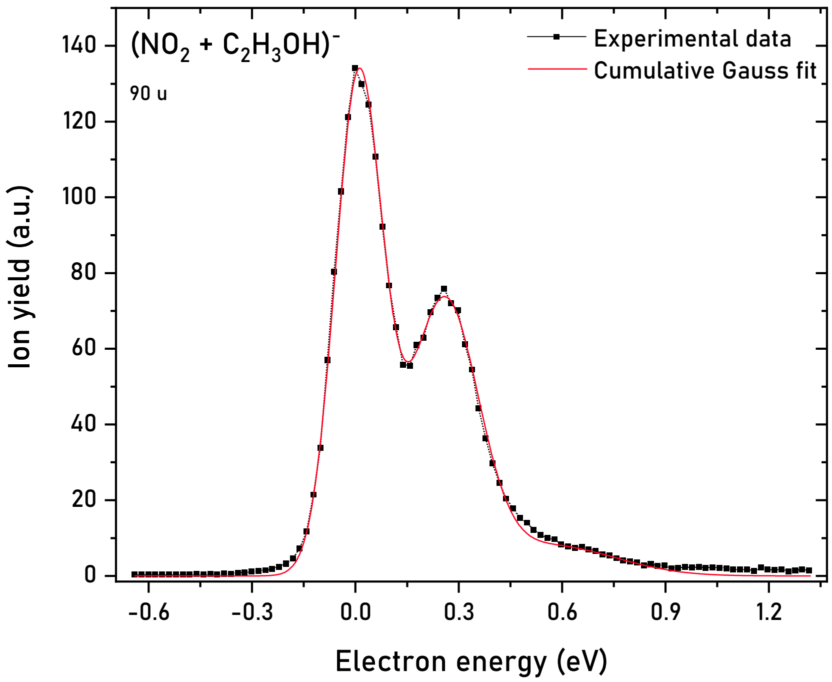


**Figure S9.** Anion efficiency curve for $(NO_2{+}C_2H_3OH)^-$ formed upon electron attachment to isolated Metro.

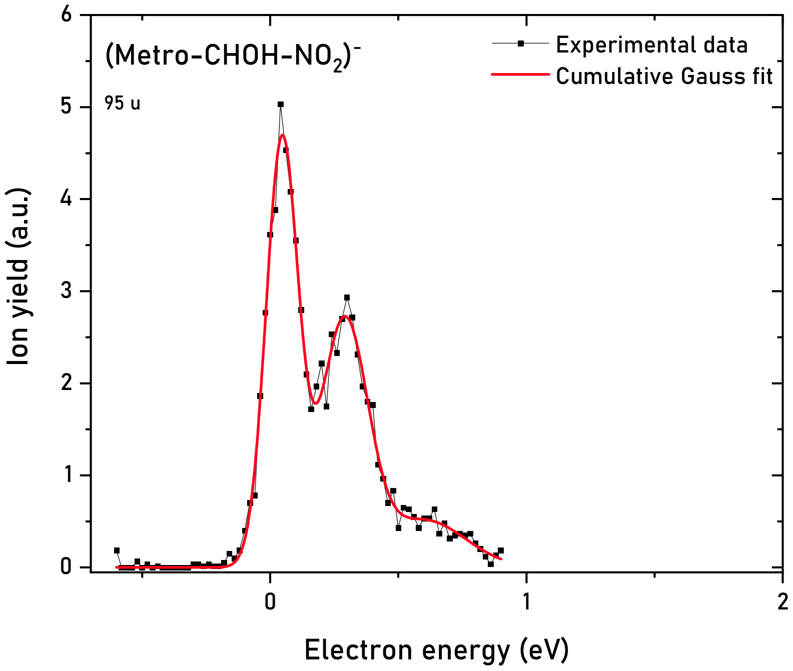


**Figure S10.** Anion efficiency curve for $(Metro\text{-}CHOH\text{-}NO_2)^-$ formed upon electron attachment to isolated Metro.

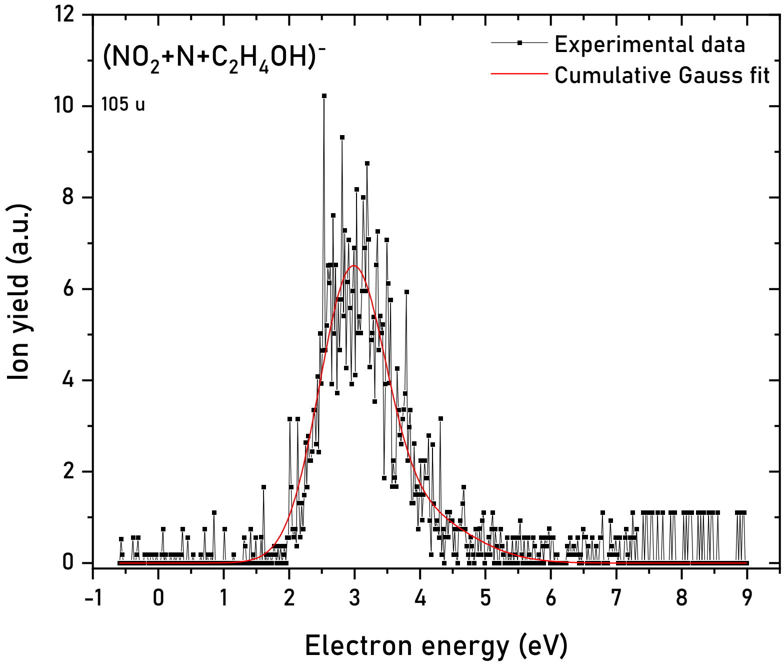


**Figure S11.** Anion efficiency curve for $(NO_2+N+C_2H_4OH)^-$ formed upon electron attachment to isolated Metro.

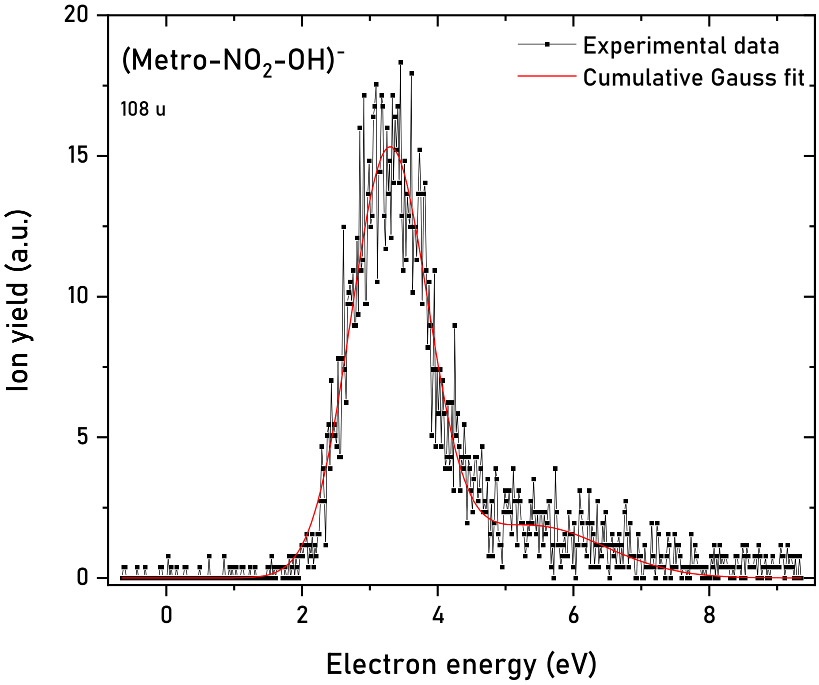


**Figure S12.** Anion efficiency curve for $(Metro\text{-}NO_2\text{-}OH)^-$ formed upon electron attachment to isolated Metro.

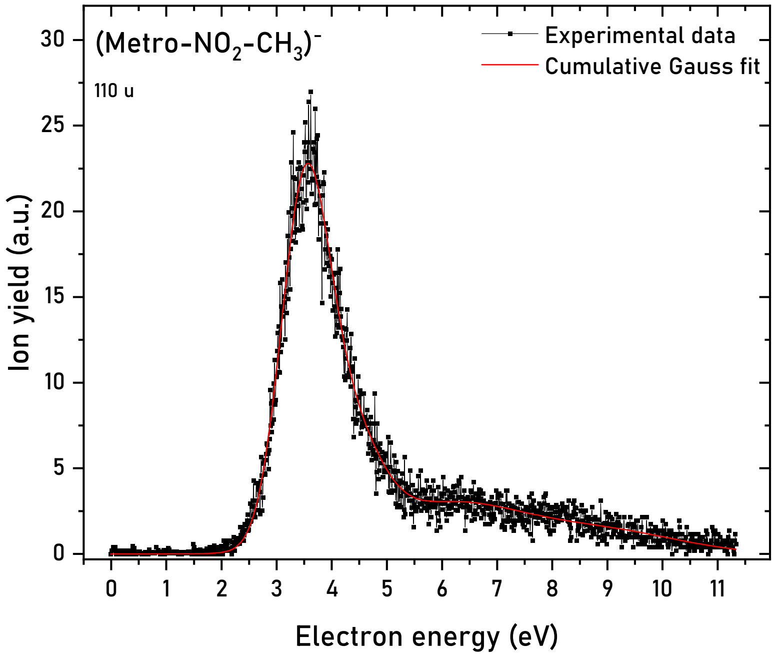


**Figure S13.** Anion efficiency curve for (Metro-$NO_2$-$CH_3$)$^-$ formed upon electron attachment to isolated Metro.

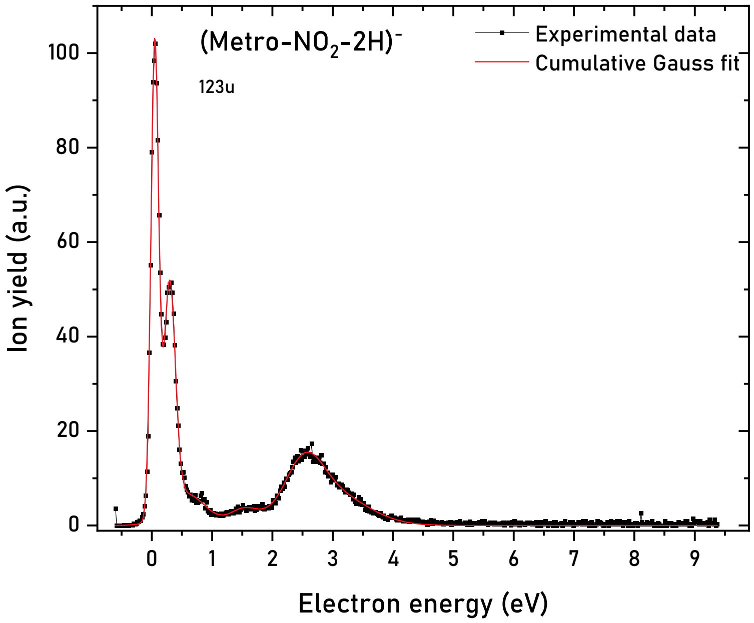


**Figure S14.** Anion efficiency curve for (Metro-$NO_2$-2H)$^-$ formed upon electron attachment to isolated Metro.

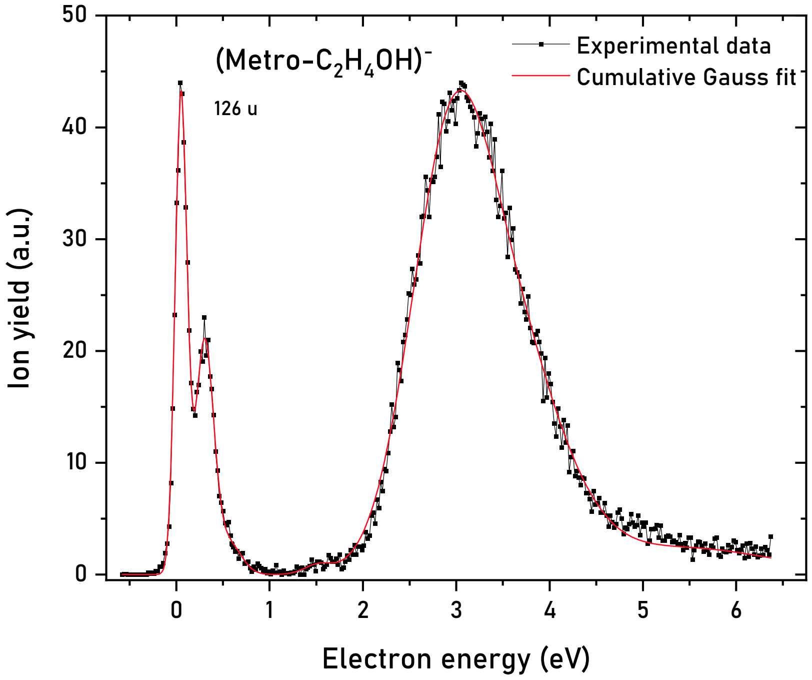


**Figure S15.** Anion efficiency curve for $(Metro\text{-}C_2H_4OH)^-$ formed upon electron attachment to isolated Metro.

XYZ coordinates for the structures shown in Figure S1-S4

**SUBSTRATE:**

**METRONIDAZOLE - NEUTRAL**

| | | | |
|---|---|---|---|
| N | 1.474617 | 5.087138 | 7.241081 |
| C | 1.017146 | 6.174539 | 6.573189 |
| C | 0.940537 | 7.535616 | 7.166151 |
| H | 1.923844 | 7.897432 | 7.468936 |
| H | 0.283632 | 7.542789 | 8.034864 |
| H | 0.540675 | 8.203825 | 6.409844 |
| N | 0.651948 | 5.884420 | 5.335095 |
| C | 0.867212 | 4.559560 | 5.180601 |
| H | 0.663993 | 4.026500 | 4.268310 |
| C | 1.369995 | 4.043513 | 6.343232 |
| C | 1.910771 | 5.063536 | 8.633641 |
| H | 2.799224 | 4.445849 | 8.715656 |
| H | 2.168448 | 6.087653 | 8.899282 |
| C | 0.819103 | 4.546756 | 9.558180 |
| H | 0.474865 | 3.576767 | 9.206061 |
| H | 1.238053 | 4.410872 | 10.557983 |
| O | -0.304206 | 5.407068 | 9.576194 |
| H | -0.166167 | 6.100704 | 10.224309 |
| N | 1.740076 | 2.696948 | 6.604969 |
| O | 1.612757 | 1.905746 | 5.695622 |
| O | 2.158315 | 2.415113 | 7.717867 |

**PRODUCTS:**

**MASS 171**

| | | | |
|---|---|---|---|
| N | 1.459824 | 5.086203 | 7.241265 |
| C | 1.000789 | 6.191327 | 6.566452 |
| C | 0.916984 | 7.547107 | 7.176695 |
| H | 1.888283 | 7.910164 | 7.523402 |
| H | 0.225777 | 7.572796 | 8.022644 |
| H | 0.549298 | 8.225602 | 6.411050 |
| N | 0.658764 | 5.907547 | 5.336797 |
| C | 0.883819 | 4.562426 | 5.177770 |
| H | 0.693132 | 4.032604 | 4.263420 |
| C | 1.377556 | 4.024757 | 6.348093 |
| C | 1.877960 | 5.051505 | 8.627919 |
| H | 2.761730 | 4.427019 | 8.710241 |
| H | 2.128534 | 6.075575 | 8.919693 |
| C | 0.811845 | 4.502374 | 9.559979 |
| H | 0.444665 | 3.563035 | 9.156015 |
| H | 1.267869 | 4.300627 | 10.535853 |
| O | -0.306480 | 5.371720 | 9.703863 |
| H | 0.015178 | 6.228622 | 9.992198 |
| N | 1.735945 | 2.729292 | 6.631879 |
| O | 1.589487 | 1.864633 | 5.703384 |
| O | 2.203879 | 2.437409 | 7.802452 |

**MASS 126**

| | | | |
|---|---|---|---|
| N | 1.304146 | 5.057226 | 7.330210 |
| C | 1.057267 | 6.136954 | 6.602579 |
| C | 0.932249 | 7.500676 | 7.202265 |

| | | | |
|---|---|---|---|
| H | 1.675410 | 8.181339 | 6.782302 |
| H | 1.074925 | 7.440966 | 8.279219 |
| H | -0.051037 | 7.928527 | 6.997197 |
| N | 0.921752 | 5.934021 | 5.261369 |
| C | 1.097438 | 4.613881 | 5.126014 |
| H | 1.056107 | 4.098902 | 4.180135 |
| C | 1.331026 | 4.078123 | 6.389629 |
| N | 1.565701 | 2.718852 | 6.689632 |
| O | 1.567821 | 1.914863 | 5.752817 |
| O | 1.757490 | 2.376991 | 7.847877 |

| | | | |
|---|---|---|---|
| C | 1.967695 | 5.131997 | 8.870562 |
| H | 2.972624 | 4.776369 | 9.036392 |
| H | 1.784044 | 5.821266 | 8.059590 |
| C | 0.815526 | 4.511313 | 9.564954 |
| H | 0.391172 | 3.694061 | 8.971273 |
| H | 1.134785 | 4.073844 | 10.517029 |
| O | -0.258596 | 5.418396 | 9.759119 |
| H | 0.087294 | 6.203778 | 10.190901 |

**MASS 123**

| | | | |
|---|---|---|---|
| c | 0.217052 | 0.021456 | -0.116963 |
| n | -0.060874 | -0.045888 | 1.226079 |
| c | 1.139419 | 0.013379 | 1.862324 |
| n | 2.149284 | 0.117165 | 1.019442 |
| c | 1.574392 | 0.116411 | -0.226921 |
| c | -1.356604 | -0.182285 | 1.829346 |
| c | -1.934035 | 0.888362 | 2.476123 |
| o | -3.027635 | 0.949153 | 3.084322 |
| c | 1.260530 | -0.039817 | 3.344967 |
| h | 0.702167 | -0.886332 | 3.744815 |
| h | 0.847017 | 0.855745 | 3.809315 |
| h | 2.313396 | -0.128168 | 3.602621 |
| h | 2.169091 | 0.189014 | -1.123136 |
| h | -1.814539 | -1.159416 | 1.768763 |
| h | -1.302245 | 1.808896 | 2.426153 |
| h | -0.579064 | 0.006535 | -0.839505 |

| | | | |
|---|---|---|---|
| n | 0.073919 | -0.000000 | 0.047505 |
| o | -0.058869 | 0.000000 | 1.220393 |
| o | 0.997313 | 0.000000 | -0.687135 |

| | | | |
|---|---|---|---|
| H | 0.000000 | 0.000000 | 0.233779 |
| H | 0.000000 | 0.000000 | 0.972811 |

**MASS 110**

| | | | |
|---|---|---|---|
| N | 1.448564 | 5.021613 | 7.306127 |
| C | 0.297511 | 5.800629 | 7.025758 |
| N | 0.080196 | 5.654608 | 5.677673 |
| C | 0.968662 | 4.767186 | 5.244923 |
| H | 0.979151 | 4.445915 | 4.209310 |
| C | 1.877540 | 4.258660 | 6.268897 |
| C | 1.958451 | 4.929423 | 8.661409 |
| H | 2.693016 | 4.124987 | 8.674022 |
| H | 2.453349 | 5.867097 | 8.931651 |
| C | 0.851383 | 4.661081 | 9.671933 |
| H | 0.335644 | 3.736187 | 9.378958 |
| H | 1.299707 | 4.496953 | 10.656225 |

| | | | |
|---|---|---|---|
| O | -0.050135 | 5.733894 | 9.783089 |
| H | -0.238184 | 5.998411 | 8.839015 |

| | | | |
|---|---|---|---|
| n | 0.073919 | -0.000000 | 0.047505 |
| o | -0.058869 | 0.000000 | 1.220393 |
| o | 0.997313 | 0.000000 | -0.687135 |

| | | | |
|---|---|---|---|
| c | 0.384284 | 0.221866 | 0.093648 |
| h | -0.083260 | -0.048070 | 1.025046 |
| h | 1.086073 | -0.449663 | -0.370722 |

**MASS 108**

| | | | |
|---|---|---|---|
| N | 1.591202 | 5.024050 | 7.155372 |
| C | 0.974854 | 6.117930 | 6.607882 |
| C | 0.803246 | 7.416467 | 7.324889 |
| H | 1.763475 | 7.872082 | 7.590271 |
| H | 0.233843 | 7.304514 | 8.252801 |
| H | 0.270403 | 8.102374 | 6.669371 |
| N | 0.591770 | 5.863320 | 5.382723 |
| C | 0.984461 | 4.544834 | 5.175575 |
| H | 0.779722 | 4.084906 | 4.218556 |
| C | 1.621437 | 3.947964 | 6.256409 |
| C | 2.065144 | 4.956535 | 8.513508 |
| H | 2.740600 | 4.094098 | 8.544863 |
| H | 2.662951 | 5.843218 | 8.758955 |
| C | 0.965545 | 4.799134 | 9.504977 |
| H | 0.081020 | 4.253810 | 9.211109 |
| H | 1.117852 | 5.034720 | 10.549647 |

| | | | |
|---|---|---|---|
| H | 1.023434 | 3.914917 | 5.174988 |
| N | 1.665335 | 2.708326 | 6.441370 |
| O | 1.628703 | 1.902831 | 5.551180 |
| O | 1.998113 | 2.577554 | 7.571551 |
| O | 1.257586 | 3.984513 | 6.113544 |

**MASS 105**

vA-vD

| | | | |
|---|---|---|---|
| h | 0.392020 | 0.108765 | -0.134252 |
| o | 0.171061 | 0.237442 | 0.799281 |
| h | 1.153680 | -0.249997 | 2.512583 |
| c | 1.399104 | 0.004006 | 1.478068 |
| h | 2.017655 | 0.905309 | 1.472007 |
| h | 1.573131 | -2.044365 | 0.901288 |
| h | 3.129133 | -1.268004 | 1.299988 |
| c | 2.164907 | -1.129874 | 0.804230 |
| n | 2.293930 | -0.890490 | -0.621018 |
| o | 4.055538 | 0.365632 | -0.036158 |
| o | 3.470590 | 0.183971 | -2.118022 |
| n | 3.286114 | -0.101327 | -0.917839 |

vA

| | | | |
|---|---|---|---|
| c | -0.046010 | -0.059778 | 0.022327 |
| c | 0.027216 | -0.046855 | 1.506426 |
| c | 1.286324 | 0.008923 | 1.913487 |
| n | 2.163773 | 0.038519 | 0.777384 |
| c | 1.428793 | -0.000908 | -0.269620 |
| h | -0.507195 | -0.963634 | -0.381896 |
| h | -0.580652 | 0.796768 | -0.394232 |

| | | | |
|---|---|---|---|
| h | 1.701307 | 0.033369 | 2.906651 |
| h | 1.858619 | 0.010601 | -1.263658 |

vB

| | | | |
|---|---|---|---|
| C | 0.658473 | 1.062549 | -0.00009000 |
| C | 1.602914 | -0.012487 | 0.00024000 |
| N | 0.563829 | -0.920209 | -0.00011700 |
| C | -0.388881 | 0.085510 | -0.00020900 |
| C | -1.855836 | -0.012167 | 0.00004900 |
| H | -2.147791 | -1.060007 | -0.00762400 |
| H | -2.281582 | 0.480721 | -0.87573400 |
| H | -2.280265 | 0.466968 | 0.88415300 |
| H | 2.662813 | -0.186643 | 0.00008700 |

vC

| | | | |
|---|---|---|---|
| C | 0.417121 | 1.054904 | -0.00017000 |
| C | -0.877647 | 0.732814 | 0.00008000 |
| N | -1.081376 | -0.695550 | 0.00003500 |
| C | 0.055367 | -1.199161 | -0.00024200 |
| C | 1.204800 | -0.228242 | 0.00008200 |
| H | 1.836083 | -0.359248 | -0.88020500 |
| H | 1.834550 | -0.359173 | 0.88155600 |
| H | -1.740220 | 1.379567 | 0.00017200 |
| H | 0.841368 | 2.045810 | -0.00027000 |

vD

| | | | |
|---|---|---|---|
| C | 0.979574 | -0.676365 | 0.00009500 |
| C | -0.422984 | -1.058706 | -0.00008200 |
| N | -1.239892 | -0.000607 | 0.00003100 |
| C | -0.424133 | 1.058238 | 0.00006500 |
| C | 0.978921 | 0.677349 | -0.00005600 |
| H | 1.820683 | 1.349296 | -0.00018600 |
| H | -0.814836 | -2.065540 | -0.00012600 |
| H | 1.822182 | -1.347302 | 0.00007700 |
| H | -0.817056 | 2.064702 | -0.00011200 |

**MASS 95**

| | | | |
|---|---|---|---|
| c | -0.145548 | -0.088311 | 0.028418 |
| n | 0.018408 | -0.061631 | 1.421054 |
| c | 1.341257 | -0.016588 | 1.765801 |
| n | 2.099294 | -0.010541 | 0.698346 |
| c | 1.190381 | -0.054364 | -0.353513 |
| c | -1.059325 | -0.079009 | 2.371622 |
| c | 1.818303 | 0.020116 | 3.179321 |
| h | 1.500128 | -0.860412 | 3.747678 |
| h | 1.447942 | 0.898014 | 3.719548 |
| h | 2.905970 | 0.052356 | 3.176544 |
| h | 1.566561 | -0.059017 | -1.367435 |
| h | -1.008650 | -0.952937 | 3.030364 |
| h | -1.061757 | 0.816683 | 3.002533 |
| h | -1.986219 | -0.115756 | 1.805141 |

| | | | |
|---|---|---|---|
| C | 0.320046 | 0.395640 | 0.358346 |
| H | 1.159727 | -0.176798 | -0.072776 |
| H | -0.097397 | 1.204027 | -0.264968 |
| O | -0.115010 | 0.151425 | 1.445262 |

| | | | |
|---|---|---|---|
| N | 0.073919 | -0.000000 | 0.047505 |
| O | -0.058869 | 0.000000 | 1.220393 |

| | | | |
|---|---|---|---|
| O | 0.997313 | 0.000000 | -0.687135 |

**MASS 90**

| | | | |
|---|---|---|---|
| c | -0.037935 | 0.376819 | 0.068341 |
| c | 0.092464 | -0.174123 | 1.442680 |
| o | 1.173778 | 0.382430 | 2.190391 |
| n | 1.001997 | 0.303644 | -0.747229 |
| o | 2.096081 | -0.210025 | -0.303519 |
| o | 0.954649 | 0.734054 | -1.935924 |
| h | -0.914481 | 0.865015 | -0.319236 |
| h | 0.227552 | -1.265038 | 1.408490 |
| h | 1.908926 | 0.302202 | 1.557894 |
| h | -0.822269 | 0.032933 | 2.000870 |

| | | | |
|---|---|---|---|
| N | 1.461534 | 5.096518 | 7.321949 |
| C | 0.989587 | 6.179598 | 6.619034 |
| N | 0.617473 | 5.914203 | 5.332529 |
| C | 0.855109 | 4.629808 | 5.201320 |
| H | 0.668493 | 4.087002 | 4.287350 |
| C | 1.387242 | 4.115956 | 6.460504 |
| H | 1.685254 | 3.103958 | 6.691168 |
| C | 0.899851 | 7.521952 | 7.223939 |
| H | 0.273761 | 7.481702 | 8.116828 |
| H | 1.890231 | 7.844134 | 7.550615 |
| H | 0.492692 | 8.234539 | 6.513968 |

**MASS 81**

| | | | |
|---|---|---|---|
| N | 1.453950 | 5.106222 | 7.318071 |
| C | 0.996847 | 6.164384 | 6.631219 |
| N | 0.629371 | 5.924102 | 5.363364 |
| C | 0.872530 | 4.587748 | 5.220104 |
| H | 0.677486 | 4.068757 | 4.291525 |
| C | 1.373874 | 4.091447 | 6.409013 |
| H | 1.673633 | 3.081369 | 6.653525 |
| C | 0.904036 | 7.530882 | 7.240491 |
| H | 0.266568 | 7.529760 | 8.128522 |
| H | 1.885846 | 7.898684 | 7.550798 |
| H | 0.487085 | 8.226015 | 6.512572 |

| | | | |
|---|---|---|---|
| C | 1.068406 | 3.967206 | 6.445577 |
| N | 1.972960 | 2.738755 | 6.520374 |
| O | 2.582722 | 2.402759 | 5.541059 |
| O | 2.022774 | 2.195398 | 7.609180 |
| C | 1.540539 | 4.993270 | 7.383898 |
| H | 1.092812 | 4.304243 | 5.416315 |
| H | 1.879129 | 5.965238 | 7.071939 |
| O | 1.539707 | 4.763240 | 8.707358 |
| H | 1.516896 | 3.808624 | 8.868624 |
| H | 0.085815 | 3.566121 | 6.710973 |

**MASS 66**

| | | | |
|---|---|---|---|
| N | 1.470481 | 5.081848 | 7.333493 |
| C | 1.014410 | 6.103516 | 6.634906 |
| N | 0.632526 | 5.931035 | 5.384536 |
| C | 0.872129 | 4.581118 | 5.223207 |
| H | 0.668661 | 4.076223 | 4.288735 |
| C | 1.378691 | 4.067877 | 6.401503 |

| | | | |
|---|---|---|---|
| H | 1.676352 | 3.054622 | 6.633487 |

| | | | |
|---|---|---|---|
| c | 0.026243 | 0.108899 | -0.022013 |
| n | 0.039520 | -0.018891 | 1.475858 |
| o | 1.061195 | 0.285718 | 2.046851 |
| c | 1.350954 | -0.330140 | -0.613751 |
| o | 1.609715 | -1.660851 | -0.455676 |
| o | -0.981523 | -0.384765 | 2.003367 |
| h | -0.170681 | 1.162685 | -0.215053 |
| h | -0.804929 | -0.497481 | -0.366102 |
| h | 2.181728 | 0.208909 | -0.134476 |
| h | 1.397949 | -0.064519 | -1.678315 |

| | | | |
|---|---|---|---|
| c | 0.384284 | 0.221866 | 0.093648 |
| h | -0.083260 | -0.048070 | 1.025046 |
| h | 1.086073 | -0.449663 | -0.370722 |
| h | 0.153617 | 1.165399 | -0.370722 |

**MASS 46**

| | | | |
|---|---|---|---|
| N | -0.042962 | 0.000000 | -0.017495 |
| O | 0.013845 | -0.000000 | 1.225248 |
| O | 1.041481 | 0.000000 | -0.626990 |

| | | | |
|---|---|---|---|
| N | 1.484040 | 5.120341 | 7.251374 |
| C | 0.994726 | 6.200115 | 6.568885 |
| C | 0.876369 | 7.561015 | 7.156833 |
| H | 1.835787 | 7.928292 | 7.524991 |
| H | 0.161180 | 7.572858 | 7.979138 |
| H | 0.526133 | 8.230806 | 6.376466 |
| N | 0.654404 | 5.871673 | 5.346276 |
| C | 0.912313 | 4.524531 | 5.200806 |
| H | 0.725444 | 3.995473 | 4.285037 |
| C | 1.413433 | 4.077079 | 6.375967 |
| C | 1.917618 | 5.035204 | 8.629736 |
| H | 2.768836 | 4.354858 | 8.678376 |
| H | 2.258391 | 6.023027 | 8.942443 |
| C | 0.815214 | 4.541845 | 9.555082 |
| H | 0.405921 | 3.612524 | 9.159568 |
| H | 1.240560 | 4.333751 | 10.541209 |
| O | -0.263967 | 5.448097 | 9.639155 |
| H | -0.012830 | 6.190229 | 10.193910 |

**MASS 42**

vA-vB

| | | | |
|---|---|---|---|
| c | -0.280757 | 0.000000 | 0.154199 |
| n | 0.420797 | 0.000000 | 1.091060 |
| o | 1.179893 | 0.000000 | 2.101407 |

vA

| | | | |
|---|---|---|---|
| O | -0.444752 | 1.314015 | 1.063912 |
| N | 0.150199 | 0.612511 | 1.950314 |
| C | 1.640363 | 0.610914 | 1.918418 |
| H | 1.991006 | 1.181097 | 2.779778 |
| H | 1.982676 | -0.419062 | 2.010354 |
| C | 2.155591 | 1.237955 | 0.638652 |
| H | 3.235715 | 1.092929 | 0.611831 |
| H | 1.718845 | 0.721628 | -0.220048 |
| H | 0.954218 | 2.710324 | 0.571294 |

| | | | |
|---|---|---|---|
| O | 1.916244 | 2.619271 | 0.587458 |
| C | -0.476914 | -0.052800 | 2.882224 |
| C | 0.259525 | -0.864415 | 3.898942 |
| H | 0.753748 | -1.726207 | 3.447602 |
| H | -0.460410 | -1.224224 | 4.627047 |
| H | 1.018138 | -0.278784 | 4.418855 |
| N | -1.850215 | 0.037550 | 2.996926 |
| C | -2.621836 | 0.507846 | 2.146416 |
| H | -3.694039 | 0.646350 | 2.302861 |

vB

| | | | |
|---|---|---|---|
| N | -0.153643 | 1.158119 | -0.00381000 |
| C | 0.587878 | 0.010631 | -0.00737400 |
| N | -0.143707 | -1.152783 | -0.00392400 |
| C | -1.382968 | -0.735830 | 0.00290500 |
| C | -1.392215 | 0.724778 | 0.00303300 |
| C | 2.062842 | 0.004959 | 0.00108000 |
| H | -2.247050 | 1.384488 | 0.00711500 |
| H | 2.419715 | -0.372113 | 0.96269300 |
| H | 2.435935 | -0.678360 | -0.76180700 |
| H | 2.450419 | 1.006194 | -0.15854300 |
| H | -2.230791 | -1.404798 | 0.00681800 |

vB

| | | | |
|---|---|---|---|
| H | 0.004119 | 1.157750 | 0.89231000 |
| O | -1.159848 | -0.246877 | 0.09454900 |
| H | -1.237070 | -0.775379 | -0.70490200 |
| H | -0.004436 | 1.157238 | -0.89287100 |
| C | -0.000014 | 0.530787 | -0.00011800 |
| O | 1.159681 | -0.247011 | -0.09456500 |
| H | 1.238806 | -0.773227 | 0.70630100 |

**MASS 26**

vA-vD

| | | | |
|---|---|---|---|
| c | 0.0000 | 0.0000 | 0.136038 |
| n | 0.0000 | 0.0000 | 1.303962 |

vA,vC,vD

| | | | |
|---|---|---|---|
| c | 0.384284 | 0.221866 | 0.093648 |
| h | -0.083260 | -0.048070 | 1.025046 |
| h | 1.086073 | -0.449663 | -0.370722 |
| h | 0.153617 | 1.165399 | -0.370722 |

vA

| | | | |
|---|---|---|---|
| h | -0.715610 | -0.023611 | -0.199829 |
| h | 0.728203 | -0.049451 | 1.815444 |
| h | 1.521132 | -0.890108 | 0.457631 |
| c | 0.681087 | -0.915394 | 1.154760 |
| c | -0.651205 | -0.911582 | 0.427824 |
| h | -0.716661 | -1.792241 | -0.219864 |
| o | -1.729941 | -0.8607 | 1.329384 |
| h | -1.618442 | -1.576394 | 1.963442 |
| n | 0.736391 | -2.105869 | 2.012678 |
| c | 1.716998 | -2.811702 | 2.091431 |
| c | 2.738620 | -3.629620 | 2.200125 |
| h | 2.854474 | -4.482282 | 1.549892 |
| o | 3.719256 | -3.431562 | 3.155585 |
| n | 4.500528 | -2.263494 | 2.870633 |
| o | 5.288330 | -2.096140 | 3.695512 |

```
vB,vD
 C     0.476219    0.000045    0.000000
 C    -0.745635   -0.000022    0.000000
 H     1.616494   -0.000141    0.000000

vB
 C      1.928259  -0.662487   0.06570900
 C      0.836835  -0.104833   0.96236400
 N     -0.287488   0.414598   0.18788000
 N     -1.282715  -0.458487  -0.11268500
 O     -1.039460  -1.6445  0.01888400
 O      2.442935   0.334946  -0.79420000
 O     -2.331518   0.009350  -0.51068300
 H      1.8116  0.486227  -1.50170300
 H      2.757533  -1.000887   0.68611200
 H      1.545522  -1.517387  -0.49471000
 H      1.232356   0.737686   1.52689700
 H      0.482046  -0.866291   1.65595800
 C     -0.641619   1.823433   0.19267700
 H     -1.098597   2.093415  -0.75423900
 H     -1.338578   2.061096   0.99886700
 H      0.283028   2.378318   0.31994200

vC
 H      2.785907  -1.526558   0.11213100
 H      1.670590  -0.068035   1.77870100
 H      0.544897  -1.229447   1.02965600
 C      1.158942  -0.345448   0.85653600
 C      2.221087  -0.649335  -0.19941300
 H      1.724821  -0.876521  -1.14743100
 O      3.144435   0.404908  -0.33510200
 H      2.654333   1.217398  -0.49154000
 N      0.413457   0.821126   0.43307300
 C     -0.789073   0.804098   0.02204700
 C     -1.531089   1.911516  -0.36384300
 H     -2.538371   2.072737  -0.70336700
 O     -2.703718  -0.497626   0.38228400
 N     -1.593569  -0.503019  -0.09074300
 O     -1.058292  -1.424946  -0.65548400

vD
 H     -1.371095  -1.408198   0.41750800
 O     -2.057161  -0.791746   0.14058700
 H     -2.278141   0.749606  -1.14077200
 C     -1.470445   0.204423  -0.65431100
 H     -0.840169  -0.223951  -1.43990200
 H     -1.301779   1.710312   0.90206200
 H     -0.223963   1.961193  -0.49512000
 C     -0.667135   1.212282   0.17373600
 N      0.416523   0.613675   0.93563000
 O      2.042760   0.374180  -0.56356300
 O      0.956264  -1.368199   0.11421200
 N      1.198581  -0.185543   0.08005200
```

**MASS 17**

```
 O     0.00  0.00  0.122270
 H     0.00  0.00  1.084321
```

| | | | |
|---|---|---|---|
| C | 1.249616 | 4.070187 | 6.452150 |
| N | 1.862376 | 2.740611 | 6.571838 |
| O | 1.633979 | 1.975526 | 5.660285 |
| O | 2.538917 | 2.494576 | 7.543435 |
| C | 1.440949 | 4.975031 | 7.386928 |
| H | 0.671528 | 4.162122 | 5.547997 |
| H | 2.049254 | 4.749048 | 8.250937 |
| H | 0.988103 | 5.950933 | 7.294867 |

| | | | |
|---|---|---|---|
| N | 1.471148 | 5.083147 | 7.300948 |
| C | 1.015521 | 6.164331 | 6.584160 |
| C | 0.947511 | 7.515859 | 7.171056 |
| H | 1.947843 | 7.838462 | 7.465666 |
| H | 0.346121 | 7.492072 | 8.081691 |
| H | 0.526060 | 8.220014 | 6.461481 |
| N | 0.641180 | 5.888284 | 5.300479 |
| C | 0.859447 | 4.598835 | 5.185839 |
| H | 0.665627 | 4.047306 | 4.278275 |
| C | 1.384209 | 4.093155 | 6.451481 |
| H | 1.6673 | 3.079851 | 6.695054 |
